# What’s Next after Hypomethylating Agents Failure in Myeloid Neoplasms? A Rational Approach

**DOI:** 10.3390/cancers15082248

**Published:** 2023-04-12

**Authors:** Hussein Awada, Carmelo Gurnari, Zhuoer Xie, Jan Philipp Bewersdorf, Amer M. Zeidan

**Affiliations:** 1 Department of Translational Hematology and Oncology Research, Taussig Cancer Institute, Cleveland Clinic, Cleveland, OH 44195, USA; 2 Department of Biomedicine and Prevention, University of Rome Tor Vergata, 00133 Rome, Italy; 3 Department of Hematology, H. Lee Moffitt Cancer Center, Tampa, FL 33612, USA; 4 Department of Medicine, Leukemia Service, Memorial Sloan Kettering Comprehensive Cancer Center, New York, NY 10065, USA; 5 Section of Hematology, Department of Internal Medicine, Yale University and Yale Cancer Center, New Haven, CT 06511, USA

**Keywords:** hypomethylating agents, myelodysplastic syndromes/neoplasms, acute myeloid leukemia

## Abstract

**Simple Summary:**

The hypomethylating agents (HMA) azacitidine and decitabine are among the standard treatment options for myeloid neoplasms. However, the treatment of patients with myelodysplastic syndromes/neoplasms (MDS) and acute myeloid leukemia (AML) who progress after treatment with HMA is challenging in the absence of standardized guidelines. Many potential novel therapeutics are under development, some of which have demonstrated promising outcomes in early clinical trials. Here we review the mechanisms and factors that predict resistance to HMA in MDS/AML patients while highlighting the latest findings in the search for therapies with significant potential in this specific setting.

**Abstract:**

Hypomethylating agents (HMA) such as azacitidine and decitabine are a mainstay in the current management of patients with myelodysplastic syndromes/neoplasms (MDS) and acute myeloid leukemia (AML) as either single agents or in multidrug combinations. Resistance to HMA is not uncommon, and it can result due to several tumor cellular adaptations. Several clinical and genomic factors have been identified as predictors of HMA resistance. However, the management of MDS/AML patients after the failure of HMA remains challenging in the absence of standardized guidelines. Indeed, this is an area of active research with several potential therapeutic agents currently under development, some of which have demonstrated therapeutic potential in early clinical trials, especially in cases with particular mutational characteristics. Here, we review the latest findings and give a rational approach for such a challenging scenario.

## 1. Introduction

The hypomethylating agents (HMA) azacitidine (AZA) and decitabine (DEC) have been a mainstay of the treatment of myeloid neoplasms for almost two decades now. These two closely-related agents primarily act by epigenetic mechanisms, the disruption of which is central to the pathogenesis of these disorders, thereby reinducing the expression of silenced critical genes [1]. In particular, HMA inhibits DNA methyltransferase-1 (DNMT-1) by forming covalent bonds between this enzyme and the DNA containing the molecules, which are incorporated during DNA synthesis [1]. HMA also possesses direct cytotoxic effects, especially at higher doses [1]. Both HMAs have been approved in oral and injectable forms by the Food and Drug Administration (FDA) for specific indications that are not interchangeable in the treatment of myelodysplastic syndromes/neoplasms (MDS) and acute myeloid leukemia (AML) [1,2,3,4]. While allogeneic hematopoietic cell transplant (Allo-HCT) remains the only potentially curative therapeutic modality for MDS, most patients are older and considered ineligible for intensive therapies such as Allo-HCT, and instead opt for lower-intensity treatments with palliative intent [5,6]. Indeed, AZA demonstrated superior overall survival (OS) vs. conventional care regimens including supportive care, and low or intensive chemotherapy in HR-MDS patients in a randomized trial setting [7,8,9]. Although DEC has not shown a similar statistically significant OS benefit versus physician choice (median OS 10.5 vs. 8.1 months, *p* = 0.38), yet it demonstrated clinically meaningful benefits such as improvements in leukemia-free survival (median 6.6 vs. 3.0, *p* = 0.004) and quality of life (QoL) endpoints [7,10]. The survival benefit of HMA is somewhat limited to HR-MDS, as neither agent has conclusive evidence of prolonging OS in low-risk LR-MDS (defined as IPSS-R < 3.5) despite increasing the hematological response and reducing the risk of leukemic progression [11,12,13,14,15]. Paralleling the observations in the HR setting, no differences with regards to the injectable type of HMA used exist also in LR-MDS (when available to be used according to local regulations) [16,17].

As for patients with AML, the use of HMA is confined to those deemed ‘medically unfit’ for intensive antileukemic chemotherapy. Medical fitness is typically determined by a comprehensive assessment that includes subjective and objective tools such as the Eastern Cooperative Oncology Group (ECOG) performance scale and Charlson comorbidity index (CCI) scores, in addition to the validated and objective Ferrara Consensus criteria [18,19,20]. Age has also often been used as a proxy for fitness, although the correlation is far from perfect. For older and unfit patients, HMA-based approaches provide an alternative, less toxic treatment options. The preference for HMA in this setting is based on several randomized trials and prospective cohorts, in which HMA led to superior clinical outcomes with limited toxicity versus other low-intensity agents such as low-dose cytarabine, other targeted therapies, or supportive care [7,21,22]. In contrast to MDS, DEC has a similar survival advantage to that of AZA in AML [23,24,25,26,27,28]. Furthermore, the enhanced survival benefits gained by combining AZA with other agents such as Venetoclax (VEN) in AML have made HMA-based combinations an even more popular therapeutic option compared to single-agent HMA [29].

Despite the encouraging evidence, long-term outcomes of HR-MDS and AML patients treated with HMA remain suboptimal, especially for patients diagnosed at an older age with 5-year survival rates not exceeding 6% [30,31,32,33,34]. Outcomes are even worse in patients who do not respond to HMA at all (i.e., primary resistance/failure) or who experience disease progression after a transient period of response (i.e., secondary resistance/failure) [35,36,37]. As such, the management of patients with HMA failure is challenging due to the aggressive nature of the disease and the lack of FDA-approved therapies, as well as the patient characteristics (elderly patients not usually fit for intensive treatments) [35]. Herein, we review the setting of HMA failure while presenting the available prognostic models that are possibly useful to predict HMA resistance, along with the latest research on novel therapeutic approaches in HR-MDS and AML patients after HMA failure.

## 2. Hypomethylating Agents Failure: Definition, Mechanisms, and Prognosis

HMA failure is generally categorized into primary or secondary based on the patient’s initial response to treatment. In MDS, primary failure is generally defined as the lack of benefit defined by blast reduction or improvement in blood counts after at least four to six cycles of initial therapy, or MDS progression to higher-risk categories or transformation to AML. Secondary failure occurs in patients who progress despite initially responding to HMA. It is defined as worsening blood counts or progression of MDS to higher-risk categories or AML following the initial response to HMA. In AML, primary HMA failure is instead defined as failure to achieve a complete remission- or complete remission with incomplete count recovery (CR/CRi) while secondary failure occurs with the loss of CR/CRi [38]. To date, studies have suggested that the primary response to HMA in HR-MDS occurs in approximately 50%, while around 36% eventually become resistant and thus develop secondary failure [36,39,40]. Indeed, long-lasting remissions are achieved in only a minority of HR-MDS and AML patients receiving HMA [7,21].

Resistance to HMA remains poorly understood with several proposed mechanisms, most likely secondary to changes in the metabolism of these drugs [41]. Indeed, HMAs are prodrugs, whose activation requires phosphorylation by the uridine-cytidine kinase (UCK) and deoxycytidine kinase (DCK) [42]. Cell-line gene knock-out experiments suggest that silencing the expression of UCK and DCK correlate with decreased AZA and DEC activities, respectively [42,43]. Valencia et al. further corroborated this finding in a study of 57 MDS patients in whom resistant patients had lower UCK expression [44]. Similarly, Wu et al. noted a significant decrease in DCK at relapse (*p* = 0.012) among MDS patients with secondary failure, while no changes were noted in those with an ongoing response [45]. Alternatively, HMAs may lose their therapeutic effect if rapidly cleared by the cytidine deaminase (CDA) enzyme. CDA catalyzes the hydrolytic cleavage which results in the deamination-mediated inactivation of HMA [46]. Mahfouz et al. studied 90 MDS patients and observed that males had higher CDA levels compared to females [47]. Higher CDA levels were associated with HMA resistance and lower survival (median 563 vs. 1033 days, *p* = 0.01) [47]. Another mechanism generating a reduction in HMA potency may be a decrease in their influx or an increase in their efflux from leukemia cells, even though reports remain conflicting as of today. Indeed, two studies have unmasked significantly higher mRNA expression of the human equilibrative nucleoside transporter (hENT1), an HMA importer, in responders vs. non-responders [45,48]. However, a third study by Qin et al. did not observe such a correlation, concluding that hENT1 downregulation does not seem to play a significant role in developing resistance to HMA [44,49]. HMA resistance through increased cellular efflux is less studied, however, in vitro models suggest that the multidrug resistance-associated protein seven (MRP7) reduces HMA accumulation in the target cells [50]. Other proposed resistance mechanisms include altered responses to DNA damage, and changes in endosomal/exosomal and microvesicular cell communication [41].

As aforementioned, HMA failure is highly associated with reduced survival. Even for LR-MDS, the post-HMA median transformation-free survival reaches only 15 months while OS barely exceeds 17 months [35]. Among HR-MDS patients, the median OS is <6 months, with only 15% alive at 2 years [36]. Factors such as increasing age, male gender, high-risk cytogenetics and mutations, bone marrow blast count, and secondary rather than primary resistance influence post-HMA outcome [36]. In contrast, undergoing subsequent Allo-HCT or using investigational agents appear to marginally improve outcomes in HR-MDS post-HMA resistance [36]. Outcomes are even worse for AML patients, who after HMA failure have a median survival between 1.3 and 2 months [36,37].

## 3. HMA Resistant MDS/AML Assessment and Prognostication Tools

Given the dismal outcomes, identifying patients at risk of HMA resistance early in the disease course is crucial. Clinical markers of prognostic utility include the presence of peripheral blasts, high transfusion burdens, and poor performance status, all of which predict resistance or a short-term response to HMA [8,51]. Conversely, an early rise in platelet counts upon HMA start reflects a return to normal hematopoiesis, potentially serving as a marker of clinical response [52,53,54,55].

The impact of the mutational landscape on HMA response in patients with HR-MDS and AML has been evaluated by Craddock et al. [56]. Multivariate analysis showed higher complete response (CR) rates in carriers of *NPM1* (OR 8.6, *p* = 0.012) mutations and lower in cases harboring *IDH2* (OR 0.4, *p* = 0.139) and *STAG2* (OR 0.3, *p* = 0.117) alterations [56]. Moreover, *CDKN2A* (median 4.5 vs. 11 months, *p* < 0.001), *IDH1* (median 5.6 vs. 11.1 months, *p* = 0.001), and *TP53* (median 7.6 vs. 11.3 months, *p* < 0.001) mutations were independently associated with decreased OS, whereas *ASXL1* (*p* = 0.035) and *ETV6* (0.033) were associated with shorter clinical responses [56]. Nazha et al. adopted a novel machine learning (ML)-based approach to identify eight mutational patterns involving *ASXL1*, *BCOR*, *EZH2*, *NF1*, *RUNX1*, *SRSF2*, and *TET2* genes [57]. These signatures were identifiable in 30% of MDS patients with ≥3 mutations and revealed a significant association with resistance to HMA and reduced OS when compared to other MDS patients with different ≥3 mutations combinations [57]. The emergence of *TP53* mutations as HMA resistance markers, including HMA/VEN combinations, was further demonstrated in the study by Schimemr et al. [58]. Using isogeneic human AML cell lines, their study showed that the synergistic effect of AZA/VEN was reduced in cell lines harboring *TP53* mutations [58]. *TP53* carriers also had lower apoptosis rates and rapid progression to AML [58].

In an attempt to simplify the prediction of HMA responses, Nazha et al. also developed a separate clinical-only ML-based model built through a multicenter effort by collecting serial complete blood counts (CBC) data from a training cohort of 424 patients who had received at least four cycles of HMA therapy [59]. The most influential parameter was a change from baseline in hemoglobin, followed by platelets, red cell distribution width (RDW), and white blood cell counts (WBC) [59]. This externally validated approach accurately differentiated patients into categories with a very low, low, intermediate, high, or very high likelihood of responses solely on variations of CBC parameters, easily trackable during patients’ follow up [59].

With regards to prognosis, the North American MDS Clinical Research Consortium scoring system was generated with the aim of predicting survival post-HMA failure with greater power than standard MDS prognostic systems, including IPSS, IPSS-R, and WPSS [60], which traditionally lack dynamism. This system classifies patients failing HMA into low- or high-risk based on a cut-off of <2.5 (median OS 11 months) or ≥2.5 (median OS 4.5 months) [60,61], resulting from the consideration of ECOG performance status (>1), complex karyotype, age (75–84 or >84 years), BM blasts (>20%), transfusion dependency, and platelet count (<30 × 10^3^/μL) [60].

Comparison of the North American MDS Clinical Research Consortium scoring system with the other conventional systems (i.e., IPSS, IPSS-R, and WPSS) asserted its greater accuracy in predicting HMA failure. Still, it remains to be explored whether the conventional system possesses a prognostic value when determined at diagnosis for HMA resistance or post-failure outcomes. In addition, more modern and mutation-driven systems have now been adopted in clinical practice. Bernard et al. proposed the molecular upgrade of IPSS, namely IPSS-M, which incorporates the aberrations of 31 genes of confirmed independent impact on MDS prognosis [62]. The prognostic advantage of IPSS-M has now been validated and challenged over IPSS or IPSS-R in external cohorts [63]. Similarly, the 2022 European Leukemia Net (ELN) recommendations incorporated mutations in *BCOR*, *EZH2*, *SF3B1*, *SRSF2*, *STAG2*, *U2AF1*, and *ZRSR2* into the adverse risk category of AML on top of their 2017’s recommendations to define cases with an MDS-like signature [64,65]. However, it remains to be explored whether HMA resistance or prognosis post-HMA failure can be predicted with the advent of these upgraded systems, and as such, studies that add these systems into the comparisons are warranted.

## 4. Management of Patients after HMA Failure

**Figure 1:**
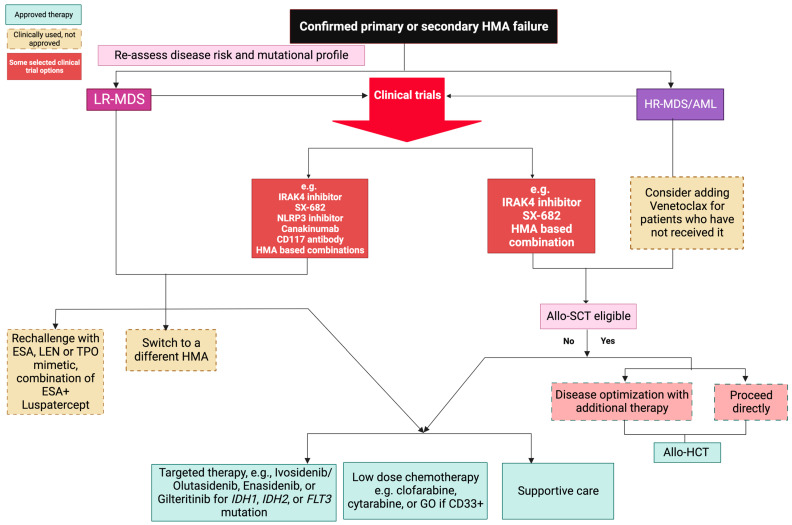
Post hypomethylating agent therapy options for LR and HR-MDS/AML. Clinical trial options are encouraged.

Management of MDS/AML post-HMA failure is complicated by the ongoing challenges in consolidating the biological and molecular heterogeneity of the disease. This includes disentangling the role of specific genetic mutations along a complex structure of gene–gene interactions [66,67]. Indeed, almost all MDS/AML patients harbor ≥1 mutation at the time of diagnosis, a situation even more complex at later stages whereby clonal evolution mechanisms may further complicate the molecular architecture [66,68]. This adds on to the extensive subclonal diversification and complexity underlying the mechanisms of resistance of primary and secondary HMA failure. In addition, most patients exhibit poor performance status, advanced age, and access to resources, all of which contribute to the overall dismal prognostic picture inherent to the biology of previously treated, advanced disease [69]. The difficulty of such a clinical scenario is substantiated by the dismal results obtained with several agents in randomized clinical trials. For instance, the phase 3 INSPIRE trial tested Rigosertib (ON-01910) in HR-MDS following HMA failure, resulting in no significant survival differences (6.4 vs. 6.3 months; *p* = 0.33) in patients treated with the experimental drug vs. physicians’ choice [70]. Rigosertib binds the *RAS* binding site of several kinases and subsequently blocks downstream cellular signaling implicated in tumorigenesis. Similarly, the use of different HMA formulations such as in the ASTRAL-3 trial using Guadecitabine showed no difference in the median OS (9.1 vs. 8.3 months in physicians’ choice group, *p* = 0.61), nor added clinically meaningful differences (secondary endpoints of 8-week transfusion independence, 12-month survival, leukemia-free survival, or duration of response in subjects with CR) [71]. Nevertheless, other alternative options and experimental therapies, are being explored and incorporated into the modern management of these patients (Figure 1) [69].

### 4.1. HMA Switch

To date, no set gold standard for second-line therapy after HMA failure has been established. The use of an alternative HMA (e.g., AZA to DEC) is not uncommon among physicians in a setting of limited options. Nevertheless, the role of sequential second-line HMA following first-line HMA failure is still controversial amid inconsistent clinical evidence [72,73,74]. This is further complicated by the poor understanding of the degree of overlap in the mechanisms of resistance between AZA and DEC [75]. The first study to assess second-line DEC included 14 AZA-exposed MDS patients, among whom a response was noted in 28% (21% CR and 7% hematological response-HR) with minimal grade 3–4 drug-related toxicities [76]. Apuri et al. reported on responses to second-line HMA in a cohort of 31 MDS patients treated at Moffitt Cancer Center. This included an overall response rate (ORR) to second-line AZA (*n* = 10) and DEC (*n* = 21) of 40% (20% CR and 20% HR) and 19% (5% CR and 14% HR) following failure of the other HMA, respectively [72]. Notably, an additional 20% of the DEC post-AZA group had stable disease and survived longer, as opposed to the other group (*p* = 0.7) [72]. Thus, sequential HMA use was initially considered a viable option in the absence of other alternatives. However, such results could not be replicated in a series by Prebet et al., whereby no response was registered in 17 MDS patients who received DEC after AZA failure [36]. Another study of 36 MDS/AML patients who failed AZA reported only a 19.4% response (8.3% CR) to DEC, all of which was short lasting and had no impact on survival. These findings were further corroborated by Duong et al. in a cohort of 25 MDS/AML patients, with no single response, the best outcome being SD in 20% and a median OS of <6 months [73]. Hence, given the small samples sizes of these studies, the absence of biomarkers able to inform on responses, and the differences in labels of AZA and DEC in HR-MDS and AML between the USA and Europe, we believe that the use of alternative HMA at the time of HMA failure may not have enough evidence to be recommended, given conflicting clinical reports and concerns for AZA-DEC cross resistance [41,77].

### 4.2. Allogenic Hematopoietic Cell Transplantation (Allo-HCT)

Allo-HCT remains the only treatment modality that holds actual curative potential in HR-MDS and AML cases [78]. Therefore, it is recommended already at disease onset to identify potential eligible candidates. This has been made feasible by the introduction of reduced-intensity conditioning regimens, which broadened the applicability of Allo-HCT also to patients previously deemed ineligible due to age and comorbidities [79,80]. While clinical trials remain lacking, evidence from cohorts reported in the literature supports the superiority of Allo-HCT to other available options in the post-HMA setting [36]. For instance, Prebet et al. showed that among 426 HR-MDS patients, those undergoing Allo-HCT after AZA failure had the highest response rate (68.4%) and longest median OS (19.5 months) as opposed to low-dose chemotherapy (0%; 7.3 months), intensive chemotherapy (13.6%; 8.9 months) and investigational agents (11.1%; 13.2 months) [36].

Current evidence suggests that exposure to prior HMA does not influence outcomes post Allo-HCT in patients with myeloid neoplasms [81]. Field et al. emphasize similar survival in AZA-exposed and nonexposed patients at 1 year post transplant (47 vs. 60%, *p* = 0.25), including those with progressive and stable disease pretransplant [81]. This is also true in AZA exposed vs. patients who received intensive chemotherapy pretransplant (57 vs. 36% at 1-year, HR 0.68 95% CI 0.35–1.3) [82]. Moreover, the sequential combination of AZA and intensive chemotherapy does not lead to significant advantages compared to AZA or chemotherapy alone in terms of OS or leukemia relapse, as shown by Damaj et al. in a cohort of 265 MDS patients [83]. However, a blast threshold of ≥5% pretransplant has been linked to worse survival (HR 2.5, 95% CI 1.3–5.0, *p* = 0.009) among MDS patients bridged to Allo-HCT with AZA [84]. Meta-analyses echo the inconclusive role of prior AZA in affecting survival (HR 0.81, 95% CI 0.6–1.0, *p* = 0.104) or relapse (HR = 0.96, 95% CI 0.7–1.3, *p* = 0.749) after transplant [85]. Altogether, these observations indicate that Allo-HCT should always be considered for eligible, HMA-exposed patients until new compelling evidence suggests otherwise. Furthermore, OS post Allo-HCT is generally longer in patients who are in CR at the time of transplant [86]. Indeed, patients who demonstrate response after four to six cycles of AZA should be considered for transplant given favorable outcomes (HR 0.22, 95% CI 0.09–0.5, *p* = 0.0001) compared to patients in PD [86]. More ongoing studies are exploring this aspect, including the ACROBAT phase 3 randomized trial (NCT04184505), testing the feasibility of up Allo-HCT upfront or versus post-AZA bridging in HR-MDS with <10% blasts as well as Allo-HCT following-HMA or 7+3 chemotherapy in HR-MDS with >10% BM blasts, though results are yet to be published. The availability of IPSS-M now as a molecular tool will further account for the molecular profiles that may help in the early identification of patients eligible and/or of favorable prognosis following Allo-HCT despite some of its limited features [87].

In addition, early studies suggested that a high-intensity myeloablative conditioning (MAC) regimen should be considered whenever clinically suitable as the risk of MDS relapse after Allo-HCT in the HMA-exposed is higher in patients who receive lower-intensity conditioning (HR 1.81, 95% CI 1.07–3.06, *p* = 0.03) [88]. Yet, recent evidence suggests that a lower-intensity regimen of Fludrabine/Treosulfan (FT) possesses survival advantages over MAC or other reduced-intensity conditioning (RIC) regimens [89]. A comparison of the three types of conditioning regimens suggested indeed that FT was associated with a lower relapse risk than RIC (HR 0.55, 95% CI 0.42–0.73, *p* < 0.001), in a similar fashion to MAC (HR 0.61, 95% CI 0.28–0.77, *p* < 0.001) [89]. In contrast, patients receiving MAC had higher nonrelapse mortality (NRM) when compared to RIC (HR 1.44, 95% CI 1.15–1.8, *p* = 0.001) and FT, which had a similar NRM risk to RIC (HR 0.88, 95% CI 0.67–1.15, *p* = 0.35) [89]. Thus, FT offers a comparable low relapse risk to MAC and a similar NRM risk to RIC, resulting in improved OS [89].

### 4.3. Low-Dose vs. Intensive Chemotherapy

Intensive chemotherapy is another option typically attempted to bridge patients with AML/MDS with progressive disease post-HMA failure to Allo-HCT. Current chemotherapy regimens that are widely used include intermediate- to high-dose cytarabine (IDAC), the 7+3 cytarabine and anthracycline course, and purine nucleoside analog (PNA)-based inductions such as fludarabine, cladribine, and clofarabine. The ORR to such regimens is not incongruent between MDS (41%) and AML (32%), with similar rates of successful bridging of responders to Allo-HCT of 40% and 42%, respectively, but longer survival among MDS patients (10.8 months vs. 6 months in AML) [90]. In a study including 307 MDS and 59 AML patients, IDAC therapy was found as an independent predictor of favorable response with ORR of 64% (OR 2.91, *p* = 0.01) vs. 7+3 (39%) and PNA (34%), while age ≥65 years was associated with lower ORR (OR 0.47, *p* < 0.01) [90]. In addition, adverse cytogenetics were associated with both lower ORR (OR 0.46, *p* = 0.01) and shorter survival (HR 1.43, *p* = 0.06) [90]. Conversely, none of the chemotherapy regimens demonstrated clinical advantage in the AML setting, albeit OS was positively influenced by anthracycline-based regimens (HR = 0.37, *p* = 0.01) and negatively affected by disease progression at the time of HMA failure (HR 2.66, *p* = 0.02) [90].

Low-intensity regimens represent a valid alternative to intensive chemotherapy in unfit cases and can be also used to induce a CR before Allo-HCT. For example, a promising low-intensity regimen consisting of intravenous clofarabine (10–15 mg/m^2^ daily for 5 days) with subcutaneous cytarabine (20 mg twice daily for 7 days every 4 to 8 weeks, and up to three cycles) can be considered in these patients [91]. In the study by Jabbour et al., 70 MDS patients underwent this regimen and achieved an ORR of 44%, with 19% of cases registering a CR and 13% bridged to Allo-HCT [91]. As with intensive regimens, complex karyotypes reduce both the response rate (OR 0.15, 0.03–0.71, *p* = 0.02) and the survival rate (HR 4.79, 2.26–10.18, *p* < 0.0001) [91].

While the introduction of a low-dose chemotherapy strategy has widened the spectrum of treatment options for medically unfit patients, still a paucity of studies exist comparing high- vs. low-intensity approaches. Current investigations are exploring the role of a fixed-dose combination of daunorubicin/cytarabine (CPX-351) in RR-MDS/AML [92,93]. CPX-351 is currently approved only in newly-diagnosed therapy-related AML or AML with myelodysplasia-related changes (AML-MRC). An early phase one trial of 43 RR-AML patients reported an ORR of 23.3% (21% CR) [92]. Another study of CPX-351, in combination with Gemtuzumab Ozogamicin (GO) in 24 RR-AML and MDS cases post-HMA failure, resulted in an ORR of 55%, though no patient transitioned to Allo-HCT due to age and comorbidities [93]. Median OS was 5 months and (95% CI 1.9–8) and median response duration was 7 months (95% CI 0–9) while no grade 3–4 nonhematological toxicities occurred [93]. However, CPX-351 does not seem to improve outcomes in newly diagnosed AML with prior exposure to HMA at the MDS stage compared to standard 7+3 chemotherapy in a subgroup analysis (HR 0.98, 95% CI 0.64–1.51) [94].

### 4.4. Targeted Therapy

**Figure 2:**
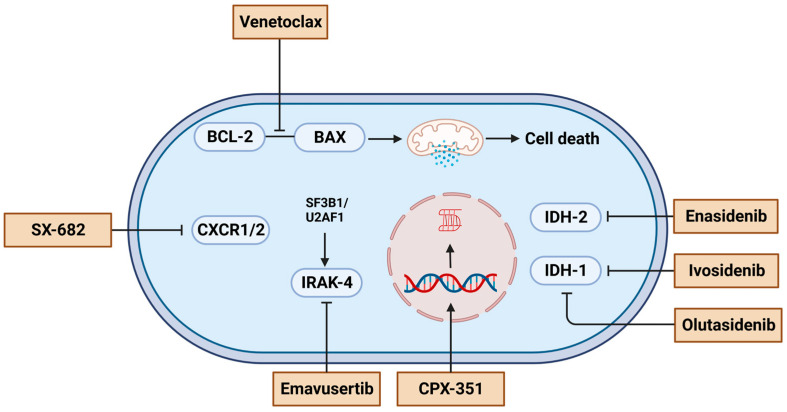
A summary of experimental agents and their cellular targets for treating myelodysplastic syndrome or acute myeloid leukemia after hypomethylating agents failure. Figures were generated with BioRender.

The improved understanding of MN pathogenesis has opened a new molecular era, enabling the identification of actionable targets and the use of targeted treatment, currently approved only for RR-AML, though not MDS. Figure 2 summarizes the list of these agents with their cellular targets. VEN, a BCL-2 specific inhibitor that promotes apoptosis, is now approved in combination with AZA/DEC or low-dose cytarabine in newly-diagnosed AML patients who are 75 or older, or who have significant comorbidities that prohibit the use of intensive chemotherapy. Yet, current evidence may encourage clinicians to use it off-label after HMA failure in both MDS and AML. A CR rate of 43% was noted in a multicenter prospective cohort of 23 patients (median age 76 years) in whom VEN was added to HMA or low-dosecytarabine after prior HMA failure, with a median OS of less than 1 year [95]. In a study by Tenold et al. comparing survival of HMA-naïve vs. exposed (median five cycles) RR-AML patients who received VEN plus HMA, a survival advantage was noticed (median OS 21.6 vs. 4.3, HR 0.411, *p* = 0.074) irrespective of ELN genetic risk [96]. In a phase 1b clinical trial of 44 RR-MDS patients undergoing VEN plus AZA, the ORR achieved was 40% (8% CR + 32% marrow CR). With a median follow up of 21.2 months, the median time to ORR and duration of response were 1.2 and 8.6 months, respectively [97]. This multicenter trial resulted in transfusion independence in 31% of previously transfusion-dependent RR-MDS patients and yielded a median OS of 12.6 months [97]. Remarkably, ORR varied widely based on baseline mutations, with the highest (83%) and lowest (20%) ORR in patients with *IDH2* and *TP53* mutations, respectively [97]. The increased sensitivity to BCL-2 inhibition in patients with *IDH1*/*IDH2* mutations confirms the results described by Chan et al.’s experiment in xenotransplanted models [98]. A similar ORR of 44% was also noted in prior studies, with no significant difference compared to HMA-naïve patients (75%, *p* = 0.82) [99]. However, these studies remain limited due to the small sample size and the absence of a definitive phase three trial, which limits the evidential bases for the use of VEN in RR-MDS/AML. As such, the risks and benefits of VEN off-label use should be thoroughly discussed with patients, and caution must be used in avoiding the extrapolation of the outcomes of newly diagnosed AML patients with relatively higher reserve hematopoiesis and less-complex disease genomics compared to RR-MDS/AML.

Targeting *IDH1* and *IDH2* mutations is currently among the available tailored approaches. The IDH2 inhibitor Enasidenib (ENA) and IDH1 inhibitors Ivosidenib (IVO) and Olutasidenib (OLU) are approved for AML though still in the experimental phase for RR-MDS. Stein et al. reported a 46% ORR (31% HI + 15% marrow CR) in the HMA-exposed (*n* = 13) vs. 75% in the HMA-naïve (*n* = 4) RR-MDS patients who received ENA in a phase 1 trial [100]. First responses were recorded at 1.2 months, with a median duration of 9 months [100]. Of note, better responses were noted in patients with a lower mutational burden [100]. After a median follow up of 11 months, the median OS in an HMA-exposed patient was noted to be the same as the entire cohort at 16.9 months [100]. Hence, the results suggested comparable outcomes in RR-MDS patients regardless of prior HMA exposure [100]. Such data are challenged by the results of the IDEAL phase 2 trial, in which HMA-exposed HR-MDS (Cohort A, *n* = 11) had a lower ORR (27%) vs. untreated HR-MDS (Cohort B, *n* = 9; ORR 56%) and ESA-exposed LR-MDS receiving ENA (Cohort C, *n* = 6; ORR 50%) [101]. Notably, 2/3 of cohort B patients who were initially resistant to ENA achieved a response after the addition of AZA [101]. OS at 1 year was lower in cohort A (55.4%) compared to B (100%) and C (80%) [101]. Thus, ENA provides a useful option in HMA-exposed MDS, though outcomes should not be expected to be as good as HMA-naive LR- or HR-diseases. A similar study design is utilized in the IDIOME phase 2 trial comparing IVO as a single therapy in the same type of cohorts, A (*n* = 13), B (*n* = 11), and C (*n* = 2), as in the IDEAL trial [102]. In this study, cohort A achieved a remarkable ORR of 54% (vs. 69% in the entire cohort), and a response was achieved in 94.4% of cases after three cycles of 500 mg per day per 28-day cycles [102]. Montesinos et al. further compared the combination of IVO and AZA versus AZA monotherapy in patients with newly-diagnosed *IDH1*-mutated AML [103]. After a median follow up of 12.4 months, IVO/AZA resulted in a significantly better event-free survival (HR 0.33, 95% CI 0.16–0.69; *p* = 0.002) if compared to AZA monotherapy, while mOS was 24 versus 7.9 months (HR 0.44, 95% CI 0.27–0.73; *p* = 0.001), respectively [103]. Cortes et al. instead reported on OLU +/− AZA in 17 MDS patients, including 11 who previously had undergone HMA use. The OLU + AZA combination group had a higher ORR of 73% vs. 33% in the OLU alone arm, with a median time to response of 1.8 vs. 4.6 months, respectively [104]. In addition, OLU appeared to have a favorable safety profile with only rare serious adverse effects; however, no differences in previous HMA exposure could be ascertained given the limited sample size [104].

Lenalidomide (LEN) represents a viable option for LR-MDS with prior HMA exposure [105,106]. Of interest, LEN post-HMA is effective even in LR-MDS patients without del(5q), resulting in a median OS of 87 vs. 107 months (*p* = 0.55), when compared to HMA-naïve patients [105]. Notably, both the standard (15 mg) and the high dose (50 mg) of LEN failed to demonstrate beneficial clinical effects in relapse/refractory HR-MDS with low response rates of 0% and 11%, respectively [106]. In addition, all LEN responders experienced grade 3/4 adverse events [106]. Alternatively, LR-MDS refractory to HMA may be treated by TGF-β pathway inhibitors such as Luspatercept and Sotatercept. HI has been reported in trials conducted by Komrokji et al. as up to 39.5% and 49% in Luspatercept- and Sotatercept-treated LR-MDS patients after HMA failure, respectively [107,108].

### 4.5. Experimental Agents

Several targeted therapies are currently studied either in preclinical or early clinical settings. Emavusertib (EMA) is a small molecule kinase inhibitor that blocks a serine/threonine kinase belonging to the interleukin one receptor-associated kinase four (IRAK4) family. Spliceosome mutations, such as *SF3B1* and *U2AF1*, induce the expression of IRAK4, leading to a targetable, longer, oncogenic IRAK4 isoform in MDS/AML [109,110,111]. Of note, IRAK4 signaling downstream of the IL-1 receptor or toll-like receptor family leads to increased NF-kB activity and promotes the dominance of MDS clones and a transition to AML [112]. EMA is currently being investigated in a phase 1 trial “TakeAim Leukemia” (NCT04278768) [71]. The study involves 49 RR-HR MDS/AML patients with prior HMA use who are receiving twice daily 200–500 mg oral EMA. Of all patients, ORR was recorded at 57% and 40% in seven MDS and five AML patients who had splicing factors mutations, respectively, while ORR was 33% in the three AML patients with *FLT3* mutations [71]. As phase 2 trials are being conducted, EMA has, thus far, demonstrated manageable safety and anticancer activity with a potential role in combination approaches in RR-HR MDS/AML [71].

During the American Society of Hematology (ASH) 2022 meeting, Sallman et al. presented the early findings of the phase 1 dose-escalation trial of SX-682 (25–400 mg twice daily) in 17 post-HMA failure MDS patients. SX-862 is an allosteric inhibitor of CXCR1 and CXCR2 chemokines, which seem to promote the growth of disease-initiating leukemic stem cells in MDS/AML [113]. All the enrolled subjects were transfusion-dependent and had failed HMA prior to the study entry [113]. The absolute neutrophil count (ANC) was used as a pharmacodynamic marker for receptor inhibition, and the dose-dependent decrease from baseline plateaued at the 200–400 mg dose range, consistent with maximum receptor inhibition [113]. This is associated with a dose-dependent increase in ORR from 0% at the 25 mg BID up to 50% at the 200 mg BID dose [113]. The 200 mg BID dose was noted to yield the most rapid and deep reduction in marrow blasts and is thus being selected for the expansion phase of the trial.

More agents remain under development as the goal is to follow the route by blocking other targetable mutations including *TP53*, *ASXL1*, *DNMT3A*, *TET2*, and others.

### 4.6. Supportive Care

Supportive care with transfusions and serum erythropoietin analogs, thrombopoietin analogs, or granulocyte stimulating factors remains a viable option for patients with low-risk disease, whose goals of care align along the palliative and comforting course rather than curative potential. Transfusions and stimulating agents are usually reserved for symptomatic patients with Hb < 10 g/dL, ANC < 1000/µL with recurrent infections, and platelets < 20,000/µL or <50,000/µL with recurrent bleeding. The aim of such options is to help lessen symptoms, improve quality of life, minimize treatment-related toxicity, and potentially lengthen survival. Symptomatic treatment does not alter the natural history of the disease, therefore, physicians should try to encourage enrollment in clinical trials for medically unfit patients to explore potentially curative therapies.

## 5. Conclusions

The best way to address HMA failure is to prevent its occurrence in the first place by improving frontline treatments and augmenting responses through HMA-based combinations. Nevertheless, targeted therapy has a promising role in the future management of MDS/AML post-HMA failure. Encouraging patients to enroll in clinical trials can help identify options with favorable therapeutic potential. Future trials specifically designed for MDS/AML post-HMA failure are warranted to help improve the current outcomes of such a challenging clinical scenario.
